# [Corrigendum] Monoamine carboxylate transporters are involved in BI-1-associated cancer metastasis in HT1080 colon fibrosarcoma cells

**DOI:** 10.3892/ijo.2026.5951

**Published:** 2026-10-19

**Authors:** Geum-Hwa Lee, Han-Jung Chae, Hyung-Ryong Kim

Int J Oncol 39: 209-216, 2011; DOI: 10.3892/ijo.2011.1024

Following the publication of the above article, a concerned reader drew to the Editor's attention the fact that five of the data panels showing the results of scratch wound assay experiments in Fig. 3C on p. 212 were strikingly similar to data that had already appeared in another paper published by the same research group in the journal *Oncogene*, albeit the images were featured in greyscale in the former paper, where the treatment conditions were reported to be different.

Upon reviewing the relevant materials, the authors have realized that errors were made in the selection of certain of the representative images shown in Fig. 3C of the above paper. A revised version of Fig. 3, showing data from an alternative set of experiments for Fig. 3C, is shown on the next page. The authors regret that this error was not identified prior to the publication of the article, and thank the Editor of *International Journal of Oncology* for allowing them the opportunity to publish this corrigendum. All authors agree to the publication of this corrigendum, and apologize to the readership for any inconvenience caused.

## Figures and Tables

**Figure 3.**
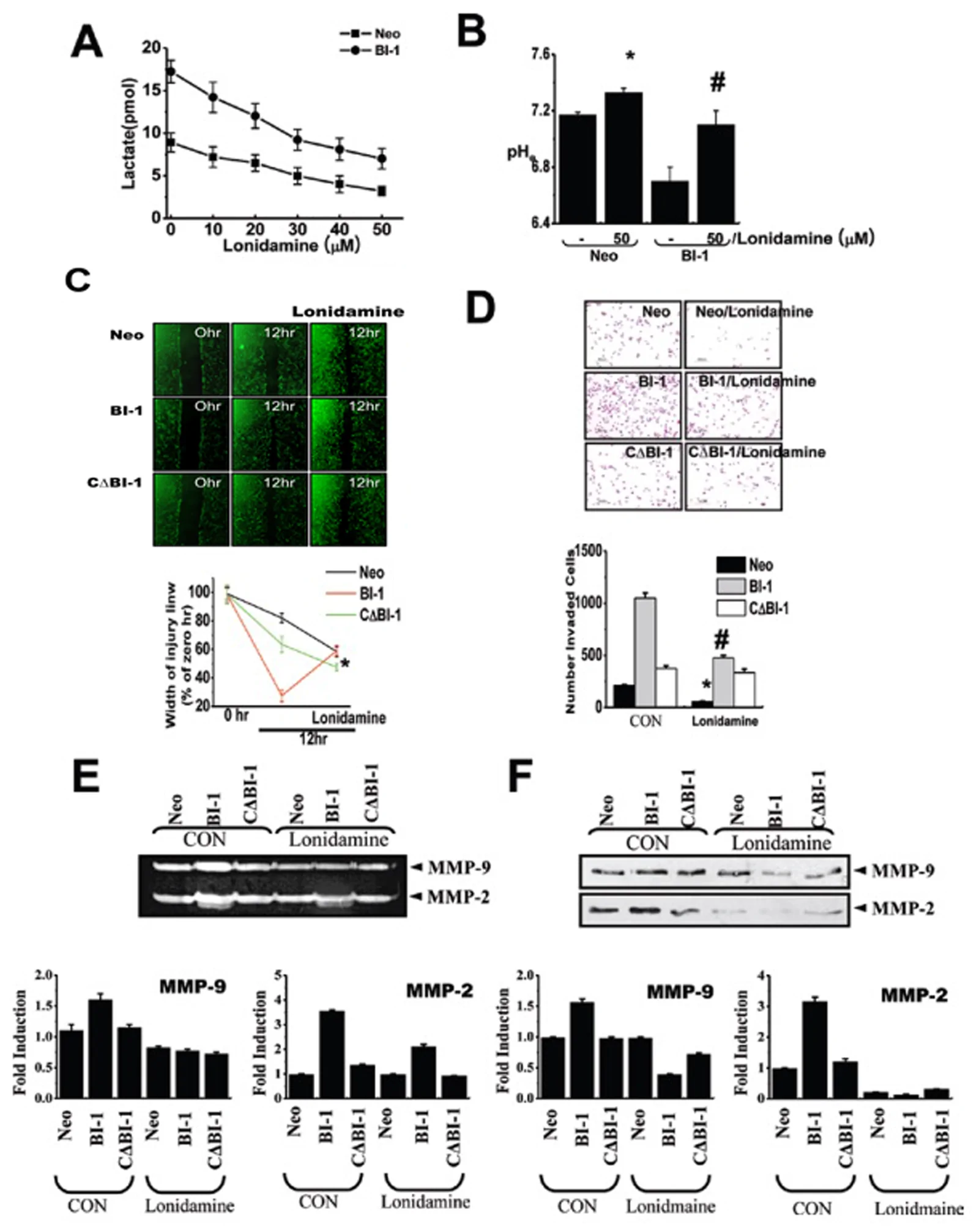
MCT activity is related to BI-1-associated cancer metastasis. (A) Neo and BI-1 cells were cultured with the indicated concentrations of lonidamine for 24 h and the MCT activity was measured as described in Materials and methods. (B) Neo and BI-1 cells were cultured with the indicated concentrations of lonidamine for 24 h and the extracellular pH was measured. (C) In Neo and BI-1 cells, an injury line was made on a confluent monolayer of cells. Cell motility was examined with a light microscope (x40) after 12 h, and the wounding width was quantified (lower). (D) In Neo, BI-1 or CΔBI-1 cells, the expression of BI-1 was confirmed (upper) and the cells were seeded onto a Matrigel invasion chamber. Samples were then incubated for 72 h in serum-free medium. The cells that invaded through the Matrigel-coated membrane were stained with 0.1% crystal violet and then examined under a light microscope (x200) (middle). The number of invading cells was determined by counting the stained cells in the lower well (lower). After Neo, BI-1 and CΔBI-1 cells were treated with 30 *μ*M lonidamine for 24 h, MMP2/9 activity (E) was measured and expression analysis (F) was performed using zymography and immunoblotting. In the lower panel, quantification was performed.

